# Mapping patient complaints to the World Health Organization patient safety rights charter: a retrospective study from Croatia

**DOI:** 10.3325/cmj.2026.67.306

**Published:** 2026-08

**Authors:** Jure Krstulović, Zrinka Hrgović, Ante Krešo, Franko Batinović, Ante Mihanović, Petra Dulčić Kardum, Ana Marušić

**Affiliations:** 1 University Hospital of Split, Split, Croatia; 2 University of Split School of Medicine, Split, Croatia; 3 Faculty of Chemistry and Technology, University of Split, Split, Croatia; 4 Faculty of Law, University of Split, Split, Croatia

## Abstract

**Aim:**

To evaluate patient complaints submitted to the Split-Dalmatia County Committee for the Protection of Patients’ Rights, classify them according to the World Health Organization (WHO) Patient Safety Rights Charter, and evaluate the impact of public awareness interventions on reporting patterns.

**Methods:**

We retrospectively reviewed 36 written patient complaints submitted from 2022 to 2024. Complaints were independently coded by two reviewers according to the ten categories of the WHO Charter. Public interventions to enhance awareness and accessibility of reporting channels included poster campaigns, media coverage, and community discussions in 2023-2024. Statistical analyses assessed temporal trends, distribution of violations, and provider categories associated with reported breaches.

**Results:**

From 36 complaints, we identified 60 WHO Charter violations, most frequently concerning the right to timely, effective, and appropriate care (38%), dignity, privacy, and non-discrimination (22%), and access to medical records (12%). Two-thirds of reported incidents involved primary care providers, while one-third involved hospital-based providers. Reporting significantly increased following visibility campaigns: charter violations increased from 11 (2022) to 36 (2024), with monthly reporting rates tripling compared with the baseline (rate ratio 3.3; 95% confidence interval 1.7-6.4; *P* = 0.001). The range of reported rights also broadened, encompassing all ten Charter categories by 2024.

**Conclusion:**

This is the first study in Croatia to evaluate systematically coded, committee-reviewed complaints according to the WHO Patient Safety Rights Charter. Our findings suggest that transparent complaint systems and sustained public education are crucial for empowering patients, strengthening accountability, and enhancing patient safety.

Patient rights are a basic pillar of modern medical systems, presenting a category of human rights established by governing authorities within the specific area of medical care (1). Legal protection of patients' rights varies among countries, generally reflecting differences in economic development, health care system capacity, and government policies (2). In the European context, the European Charter of Patients’ Rights (ECPR) serves as a foundational document that outlines 14 fundamental patients’ rights. Drafted in 22 languages, it has become a key reference for European Union (EU) citizens’ rights in health care and a milestone in the development of subsequent EU charters (3). In 2024, the World Health Organization (WHO) launched the Patient Safety Rights Charter as an integral document that supports the Global Patient Safety Action Plan 2021-2030 (4). It seeks to eliminate harm in health care by empowering patients and promoting fairness, integrity, access to data, and risk reduction as essential tenets of safe and quality care (4). The WHO Charter expands previous regional frameworks, such as ECPR, by emphasizing patient safety and striving for consistent protections across various global health care systems (5). The document outlines 10 patient rights (Table 1) (4).

**Table 1:** Matching the reported patient right violations (n = 60) to the World Health Organization Patient Safety Charter violations

Charter right	No. (%)
Timely, effective and appropriate care	23 (38)
Dignity, privacy and non-discrimination	13 (22)
Access to medical records	7 (12)
Safe and rational use of medicines/products	5 (8)
Information and decision-making support	3 (5)
Be heard and fair resolution	3 (5)
Safe care processes and practices	2 (3)
Qualified and competent health workers	2 (3)
Safe and secure facilities	1 (2)
Patient and family engagement	1 (2)
**Total**	**60** (100)

An effective method to examine patient needs and their rights within the system of health care is the review of patient claims (6). Studies from European countries show that patient complaints offer important insights into the factors that cause medical mistakes, which are essential to strengthening the safety of patients (7,8). Just as European countries have different legislation in protecting patients' rights, they also employ a variety of governmental and institutional bodies, ombudspersons, or other methods to address patient complaints, or court systems (9). Nevertheless, there is a lack of research explicitly investigating the function and efficacy of regional or local committees and other entities responsible for empowering patients about their rights and enabling access to complaint processes.

The Global Patient Safety Action Plan also aims to establish patient safety reporting systems that encourage patients and families to report incidents of unintended or unnecessary harm that may have been contributed by health care practices (10). Despite the growing global initiatives to improve patient safety, more than 6% of patients experience preventable harm during health care, with a significant percentage of these occurrences leading to permanent disability or mortality (11). By collecting, organizing, and analyzing these patient-reported experiences and outcomes related to unsafe care, the Global Patient Safety Action Plan 2021-2030 aims to identify opportunities for learning and improvement, ultimately supporting the development of highly reliable health care systems and institutions that protect patients daily from violations of their rights (12).

The Croatian Health Insurance Act ensures universal health care through mandatory insurance based on equality and reciprocity. The main goal of the Act is to ensure equal, comprehensive care that safeguards patients' rights and safety (13). Croatia's health care system has three levels: primary care (mainly by family physicians, pediatricians, and gynecologists), secondary care (specialist outpatient services in health centers, polyclinics, and hospitals), and tertiary care (highly specialized services in clinical centers or national institutions). The Ministry of Health oversees legislation, planning, administration, and management of publicly funded health care institutions (14).

In accordance with the Croatian Act on the Protection of Patients’ Rights (PRPA) (15,16), each county is legally obliged to establish a Committee for the Protection of Patients’ Rights, which serves as an expert advisory body to the County Assembly and operates under the PRPA (17). Its duties include receiving and investigating citizens’ complaints, determining whether patients’ rights have been violated, monitoring the implementation of relevant regulations, and reporting serious violations to the Ministry of Health. The Committee reports violations impacting many patients. It follows the County Assembly's Rules of Procedure (18). Members are chosen through a public call, representing patients, NGOs, and experts.

Although the WHO Patient Safety Rights Charter provides a comprehensive global framework for patient rights, evidence regarding its practical application in real-world health care systems remains scarce. In particular, no previous study has evaluated whether officially submitted patient complaints can be systematically classified using the Charter or whether it can serve as a standardized framework for analyzing patient rights violations within an established legal reporting system.

The aim of this study was to match complaints filed by patients about the violation of their health care rights sent to the Regional Patient Rights Protection Committee of Split-Dalmatia County with the WHO Patient Safety Rights Charter. We also evaluated how different target activities of the Committee – public outreach campaigns, educational programs, and intersectoral collaboration with health care institutions and civil society organizations – contributed to raising awareness of patient rights, as reflected in the use of available channels for reporting their violations.

## METHODS

### Study design

We retrospectively reviewed the patient complaints sent to the Committee for the Protection of Patients’ Rights of Split-Dalmatia County from 2022 to 2024 via a formal email.

### Promotion interventions

**Figure 1:**
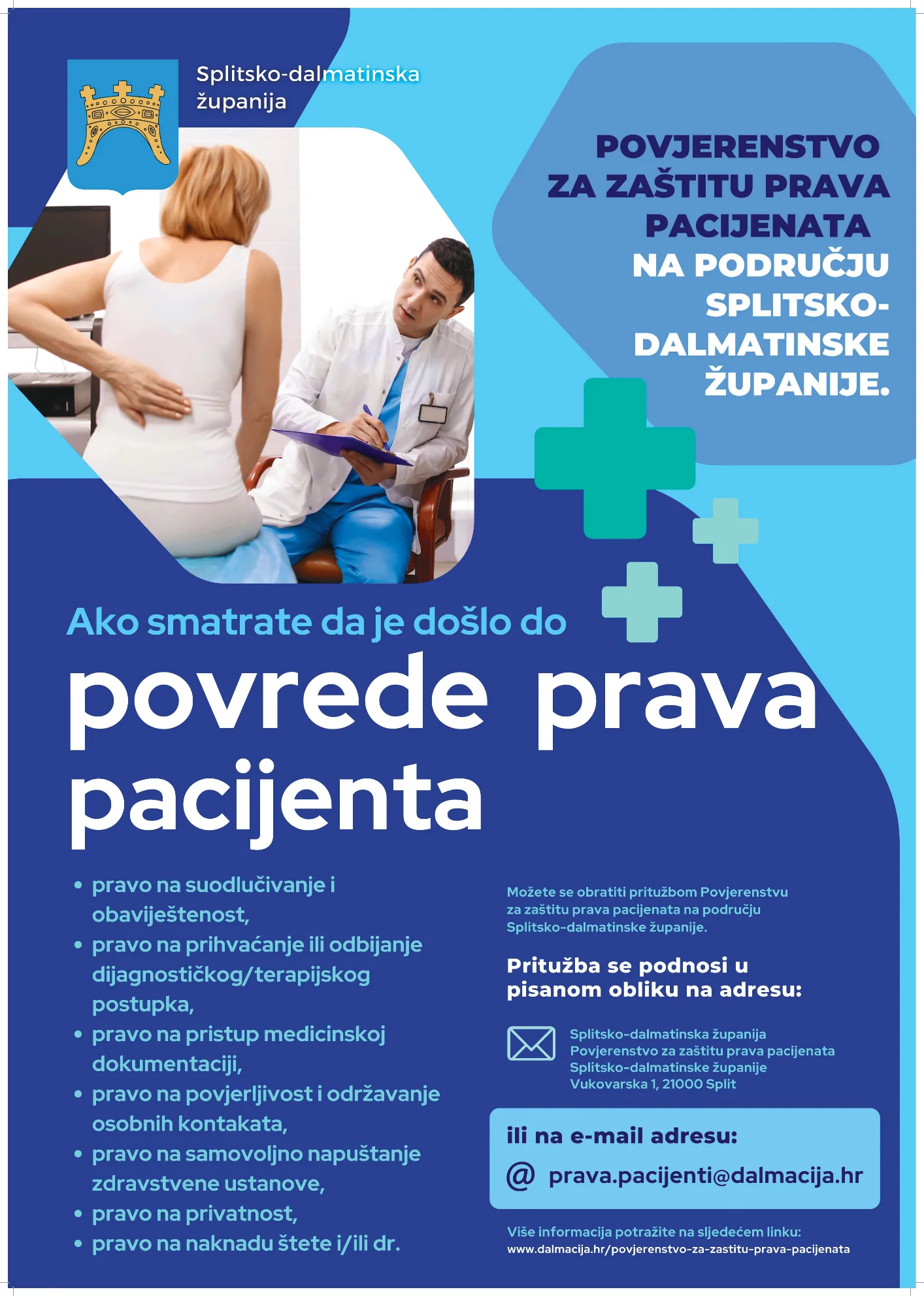
A poster promoting patient rights and the procedure for submitting patient complaints.

To promote awareness of patient rights, we implemented several outreach activities (Table 2). In 2023, we launched a seven-event outreach program, distributing 227 posters (Figure 1) outlining the WHO Charter of Patient Rights across various institutions: the Health Center of Split-Dalmatia County (100 posters), the Teaching Institute of Public Health of Split-Dalmatia County (45 posters), the Emergency Medical Service of Split-Dalmatia County (45 posters), the Croatian Health Insurance Fund (13 posters), and the Croatian Pension Insurance Institute (13 posters); 11 posters were given to social care and non-governmental organizations.

**Table 2:** Outreach activities employed to increase awareness of patient rights and the official complaint mechanism

Year (campaign phase)	Outreach events (n)*	Representative activities	No. of identified World Health Organization Charter violations
**2022 – pre-campaign**	0	–	11
**2023 – early campaign**	7	Online and radio interviews; two interviews in the local daily, *Slobodna Dalmacija*, RTL, and Nova TV coverage of a high-profile case; committee website; “Patients’ Rights” posters designed	13
**2024 – late campaign**	7	Posters installed county-wide and media bundle implemented (TV stations: HRT 1, Al Jazeera Balkans); daily *Slobodna Dalmacija* print and web); *Sunce* Radio; RTL TV report (a case study of an immobile patient transported to a wrong address); participation in the European Medical Students’ Association Split panel discussion entitled “Strike in Healthcare: Ethical Dilemmas on Patients' Rights” with participation of experts from medicine, law, and patient advocacy.	36

*Multiple appearances at the same medium were counted as one event.

### Ethical considerations

In accordance with Article 33 of the Croatian Act on the Protection of Patients’ Rights, these reports are submitted annually to the County Assembly and the Ministry of Health. Following their adoption by the County Assembly, they become publicly available documents under the Croatian Act on the Right of Access to Information. No identifiable personal data were accessed or processed, and all analyses were performed on anonymized information contained in these public reports. Therefore, in accordance with Croatian legislation and institutional regulations, formal ethics committee approval was not required. Two authors (JK, PDK), who serve as president and vice-president of the Committee, independently classified the anonymized complaints according to the WHO Patient Safety Rights Charter. Any disagreements were resolved through discussion, and unresolved cases were adjudicated by the remaining authors (ZH, AK, AM) using anonymized complaint summaries only.

### Statistical analysis

We considered each report a distinct violation for each right. Patient complaints were classified by analyzing completely anonymized information given by the Committee President. The number of complaint letters (n = 36) annually was used for time-trend analyses, while individual WHO-charter violations (n = 60) provided categorical detail for cross-sectional comparisons. Because a single complaint could include more than one Charter violation, coded violations were treated as descriptive classification units rather than statistically independent patient observations. Each violation was further classified by provider category using a standardized coding structure with 13 mutually exclusive groups.

The results are presented as counts (percentages) and medians with interquartile ranges. Associations between categorical variables, specifically (i) charter rights by year, (ii) charter rights by gender (pooled across years), and (iii) provider category by year were assessed using Pearson χ^2^ test. Rows containing only zero counts were omitted in advance to meet the minimum expected-cell requirements. All tests were two-sided, with *P* values <0.05 considered statistically significant. The statistical analysis was conducted with R, version 4.3, including the *epitools* and *stats* packages (R Foundation for Statistical Computing, Vienna, Austria).

Yearly complaint counts were standardized to “complaints per month” by dividing by 12 months. Temporal trends across the three-year period were evaluated using simple linear regression, with year coded as 0, 1, and 2. Differences in complaint rates between years were further summarized using Poisson rate ratios with 95% confidence intervals calculated with standard large-sample methods.

Given the exploratory, hypothesis-generating nature of the study, no adjustment for multiple comparisons was applied. Exact *P* values are reported to enable readers to interpret the strength of evidence. All analyses followed the STROBE guidelines for observational studies.

## RESULTS

### Complaints profile 2022-2024

We identified 60 distinct WHO-charter violations in 36 written complaints. Most of the violations (n = 41, 68%) were reported by female complainants. The letters contained a median of one violation (IQR 1-2). Health provider details were provided in all complaints. Among 37 provider mentions (one letter named two services), incidents most frequently involved primary-care physicians (15/37, 40%) and hospital specialists (11/37, 30%). Other providers were less frequently mentioned: private practices (3/37, 8%); dental medicine, laboratories, and physiotherapy and nursing/technician services (2/37 each, 5%). Primary settings accounted for 24/36 (67%) complaints, and hospital-based care for 12/36 (33%). No secondary level care cases were registered.

### Types of rights violations

The complaints matched all ten WHO Charter rights (Table 1). The most frequently violated right was the right to timely, effective, and appropriate care (23/60, 38%), followed by the right to dignity, privacy, and non-discrimination (13/60, 22%). Violations of access to medical records were the only other category above 10% (7/60, 12%).

### Annual trends

WHO charter violations rose markedly over the three years: from 11 in 2022 to 13 in 2023, and then 36 in 2024. Thus, violations reported in 2024 alone generated 60% (36/60) of all the violations identified in patient complaint letters. The number of patient complaint letters also increased, from 4 in 2022 to 8 in 2023 and 24 in 2024.

Using 2022 as the baseline, the monthly reporting rate increased from 0.9 to 1.1 in 2023 and 3.0 violations in 2024 (Table 3). The early-campaign rise was non-significant, but the late-campaign rate was almost three times higher than baseline (*P* = 0.001) or the early-campaign level (*P* = 0.002).

**Table 3:** Monthly rates and Poisson rate ratios of reported patient rights violations*

Period	Complaint letters (n)	Violations	Months	Monthly rate	Rate ratio vs previous (95% confidence interval)	*p*-value^†^
Pre-campaign 2022	4	11	12	0.92	-	-
Early-campaign 2023	8	13	12	1.08	1.2 (0.5-2.6)	0.68
Late-campaign 2024	24	36	12	3.00	2.8 (1.5-5.2)	0.002
Late 2024 vs Pre 2022 campaign	—	—	—	—	3.3 (1.7-6.4)	0.001

*Rate ratio confidence intervals were calculated as RR × exp [±1.96 × √(1/ count_1_ + 1/ count_2_)] for Poisson counts with equal exposure.

†Wald test on the natural logarithm of the Poisson rate ratios.

**Figure 2:**
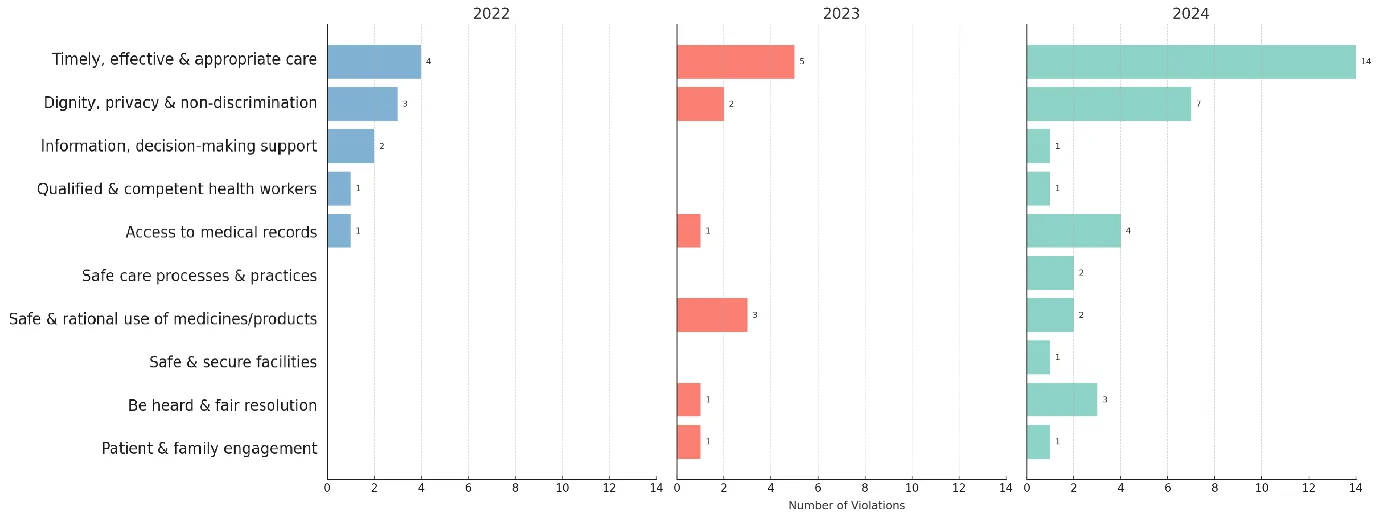
World Health Organization Charter Right violations reported by patients, 2022-2024.

Over the three-year period, the complaint profile broadened markedly: the number of violations that were already present in 2022 (chiefly timely care and dignity/privacy) increased year on year, while violations of additional rights such as access to medical records, safe use of medicines, safe care processes, safe facilities, fair resolution, and patient-family engagement began to appear for the first time. This finding indicates both a higher volume and a wider spectrum of patient-reported WHO-charter infringements (Figure 2).

**Figure 3:**
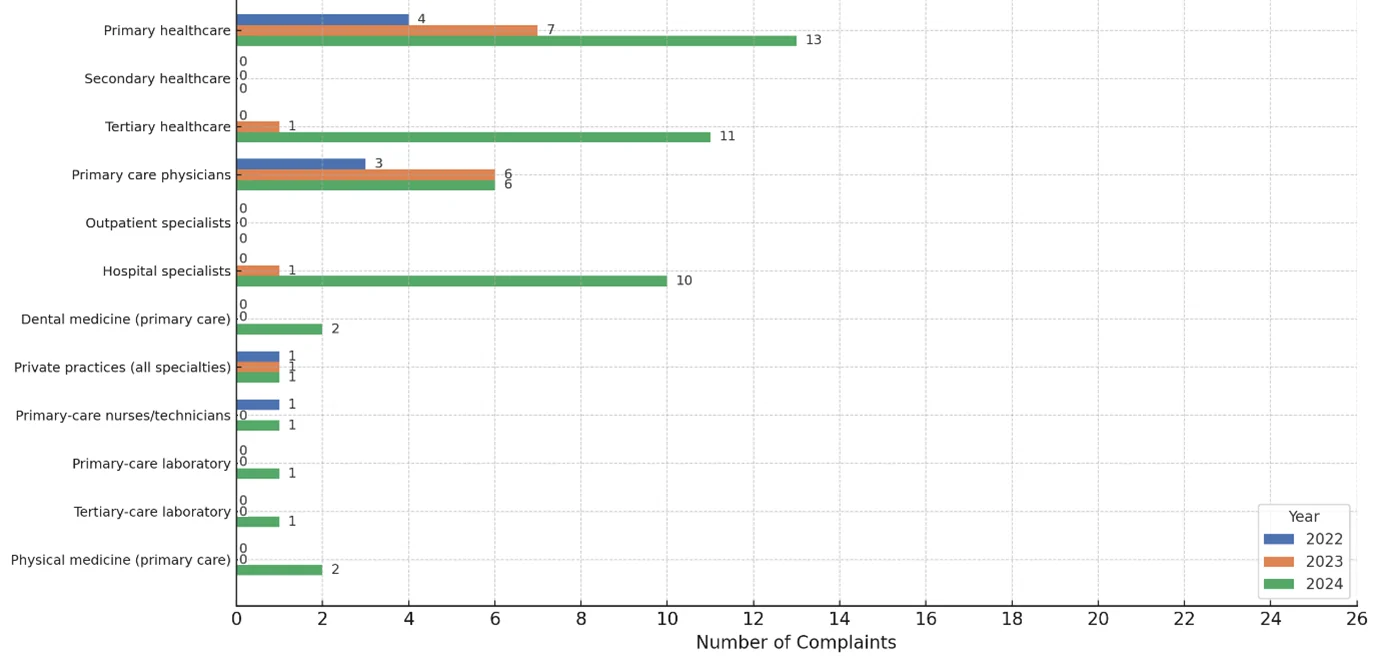
Reported World Health Organization Charter violations by health care institution type, 2022-2024.

The complaints (Figure 3) were limited to isolated incidents at hospital-based care and individual specialists in 2022, but also involved primary-care physicians, outpatient specialists, and community health centers in 2023. The complaints surged in 2024 to cover all WHO Charter violations – reaching double-digit counts for hospital specialists (n = 10), hospital-based care (n = 11), and primary-care facilities (n = 13).

## DISCUSSION

To the best of our knowledge, this is the first research to longitudinally assess patient complaints according to the WHO Patient Safety Charter (4) as a coding framework. Unlike a previous report that relied on non-governmental complaint sources (19), our analysis captured systematically logged, committee-reviewed complaints submitted in writing, thus offering a unique, granular view of rights violations as perceived by citizens within the official health system to a county-level committee.

During the study period, we observed an increase in the number of reported patient complaints and identified rights violations. This increase should be interpreted within the broader context of national trends in Croatia, increased public awareness activities, and the existing organizational pathways for handling patient complaints. Although the absolute number of complaints remained modest (36 over three years), the relative increase – from 4 to 24 letters annually, and from 11 to 36 rights violations – represents a nearly 3-fold rise year-on-year. Importantly, the breadth of the increase suggests a generalized reporting effect rather than a concentration in any specific patient group, provider category, or type of right violated.

The most frequently violated right in our study was the right to timely, effective, and appropriate care, followed by the right to dignity, privacy, and non-discrimination. These categories align with prior findings from both Croatian and international contexts (19-21), underscoring persistent systemic issues within the public health care system, particularly regarding accessibility, waiting times, and the perceived quality of interpersonal communication in health care delivery. Access to medical records, ranking third, may reflect ongoing difficulties with health information transparency despite legal provisions under Croatian and EU frameworks (22). Croatia has implemented electronic health records (eKarton) in hospitals, with information accessible to patients through the e-Citizens (e-Građani) portal, which enables viewing of diagnostic test results, prescriptions, chronic conditions, vaccination records, and more (23). However, digital health literacy remains low, with a recent national survey showing that insufficient or inadequate health literacy is linked to poorer access to care, including unmet medical needs due to long waiting times (24). This suggests that while electronic patient record systems exist, many patients struggle to effectively navigate and understand them.

Although other research is dominated by complaints regarding hospitals and tertiary institutions (25), in our study, about two thirds of the complaints originated from primary care settings (67%), with hospital-based care accounting for the remaining third. This may be attributable to the Committee’s greater visibility and accessibility at the community level, as the posters were placed in primary health care facilities throughout the county. Unlike earlier studies that showed greater distrust in hospital-based care (25,26), the lower proportion of complaints from hospital-based care in our study is likely due to the hospitals’ established complaint management system.

Croatia lacks a standardized procedure or pathway for addressing patient complaints. A patient has the option to file a complaint both separately and concurrently with multiple entities, including the health care institution involved, the Ministry of Health, regional committees for the Protection of Patients’ Rights (26), and the Ombudsperson of the Republic of Croatia. Recent annual reports of the Ombudswoman of the Republic of Croatia indicate a continuous increase in complaints related to health care and difficulties in exercising patients' rights nationwide (27). Therefore, the trends observed in Split-Dalmatia County should be interpreted within the broader Croatian context rather than being attributed solely to local awareness activities. Healthcare providers typically serve as the initial point of contact for patient complaints (28). Every health care institution is required to have an internal complaint management system, including mechanisms for addressing patient concerns through its Health Care Quality Commission (29). These internal pathways may allow complaints to be resolved at the institutional level before they are submitted to the county committee. Consequently, the relatively lower proportion of complaints concerning hospital care received by the county committee should not necessarily be interpreted as indicating fewer patient rights violations in hospital settings. Patients can complain concerning the behavior of health professionals to their respective professional chambers, including the Croatian Medical Chamber or the Croatian Chamber of Medical Nurses. These professional organizations oversee the safeguarding of patients’ rights from the perspective of professional ethics and deontology (30). Outside the institutional framework, the Croatian Association for the Protection of the Patients’ Rights is a non-profit, non-government organization that provides direct legal and expert advice to the patients (31). Lastly, patients have the option to submit complaints against the Republic of Croatia directly to the European Court of Human Rights (32).

In our study, gender differences were not found to be significant, nor was there a variation in rights-violation patterns by year or type of provider. The increase in complaints in 2024 is likely related to improved visibility and easier access to reporting following the implementation of an integrated campaign. Media coverage and committee activities were not external confounding factors but deliberate components of the intervention designed to increase awareness of patient rights and improve access to the official reporting pathway. Although this temporal association should not be interpreted as evidence of causality, it is consistent with the intended mechanism of the intervention. The observed increase may also be affected by other factors, including national trends in patient complaints. A rate-ratio analysis confirmed a significant increase in 2024 compared with 2023. The results emphasize the importance of visibility and accessibility in the complaints process. Prior studies have shown that low trust in public institutions and a lack of procedural clarity can suppress reporting (33,34). In contrast, our data suggest that enhancing patients’ awareness of the mechanisms for asserting their rights may empower them to voice concerns, potentially serving as an indirect measure of system responsiveness.

Our study has several limitations. First, its retrospective design precludes causal inference. Second, the data set covers a single Croatian county and consists only of written complaints. Third, as data were analyzed in aggregate, we could not explore individual trajectories, such as whether repeat complainants or complex cases contributed disproportionately to the 2024 spike. Finally, the relatively small sample size and exploratory nature of the analyses warrant cautious interpretation of the findings.

Future research should investigate whether the increase in complaint rates is accompanied by improvements in institutional response, transparency, or corrective actions. Educational efforts to raise awareness of patient rights both among health care professionals and the public may also be warranted, particularly in primary care settings, where most violations were observed.

In conclusion, a sharp increase in reported rights violations in 2024 coincided with the rollout of a county-wide visibility campaign, which suggests that the primary drivers of the increase were improved awareness and access, while broader national trends may have contributed to a lesser extent. Most complaints originated from primary care, likely due to targeted poster placement, while the absence of complaints from tertiary care may reflect hospitals’ internal complaint systems. The most frequently reported violations involved access to timely and effective care, dignity, and privacy, and the right to access medical records. Although limited by aggregate data and geographic scope, the findings highlight the importance of transparency, patient education, and simple reporting systems that help patients speak up and hold the health system accountable.
